# Geographic disparities in the risk of perforated appendicitis among children in Ohio: 2001–2003

**DOI:** 10.1186/1476-072X-7-56

**Published:** 2008-11-04

**Authors:** Robert B Penfold, Deena J Chisolm, Benedict C Nwomeh, Kelly J Kelleher

**Affiliations:** 1 The Research Institute at Nationwide Children's Hospital, Center for Innovation in Pediatric Practice, 700 Children's Drive, Columbus, OH 43205, USA; 2 Division of Pediatric Surgery, Nationwide Children's Hospital, 700 Children's Drive, Columbus, OH 43205, USA

## Abstract

**Background:**

Rural-urban disparities in health and healthcare are often attributed to differences in geographic access to care and health seeking behavior. Less is known about the differences between rural locations in health care seeking and outcomes. This study examines how commuting patterns in different rural areas are associated with perforated appendicitis.

**Results:**

Controlling for age, sex, insurance type, comorbid conditions, socioeconomic status, appendectomy rates, hospital type, and hospital location, we found that patient residence in a rural ZIP code with significant levels of commuting to metropolitan areas was associated with higher risk of perforation compared to residence in rural areas with commuting to smaller urban clusters. The former group was more likely to seek care in an urbanized area, and was more likely to receive care in a Children's Hospital.

**Conclusion:**

To our knowledge, this is the first study to differentiate rural dwellers with respect to outcomes associated with appendicitis as opposed to simply comparing "rural" to "urban". Risk of perforated appendicitis associated with commuting patterns is larger than that posed by several individual indicators including some age-sex cohort effects. Future studies linking the activity spaces of rural dwellers to individual patterns of seeking care will further our understanding of perforated appendicitis and ambulatory care sensitive conditions in general.

## Background

Appendectomy for appendicitis is one of the most frequent surgical procedures performed in the United States [1,2]. Children are at the highest risk of perforation and perforated appendicitis is associated with $1.5 billion in annual healthcare charges [1]. On average, 35 percent of appendicitis cases are "ruptured" or "perforated" prior to surgery, increasing both cost and risk of poor surgical outcome [3].

Perforated appendicitis is considered an ambulatory care sensitive condition (ACSC) that can be prevented with timely and appropriate care [4]. It has been used as a measure of access to preventative care [5]. The Agency for Healthcare Research and Quality has called for research regarding the relationship between geographic factors and hospital admission for ambulatory care sensitive conditions at the ZIP code level [4]. To date, such research has primarily focused on rural-urban comparisons and not sufficiently examined intra-rural variation. This paper uses intra-rural RUCA codes (Rural-Urban Commuting Areas) [6] to differentiate rural locations at the ZIP code level and examine geographic variation in the risk of perforated appendicitis among children in Ohio. This geographic approach has implications for the location of healthcare services because farmers (particularly small farm operators) are increasingly working off-farm and therefore commuting to work [7]. Commuting patterns may be associated with new patterns of health care seeking to the extent that individuals find it more convenient to seek care near work locations.

A number of clinical and demographic factors are known to be associated with a higher risk of perforation [8-10]. Parental delay in seeking medical attention is one of the strongest factors associated with perforated appendicitis among children [11]. Although a recent study of perforated appendicitis in children found that hospital location (urban versus rural) and hospital type (teaching versus non-teaching) were not significantly related to higher perforation rates [9], there is significant geographic and practice setting variation in the management of acute appendicitis [12]. It is likely that intra-rural variation may have contributed to the failure to identify location effects in previous research.

Studies of geographic variation in risk and health care outcomes have tended to use simple distance metrics in order to measure the effects of location [13]. However, distance and travel time do not measure important characteristics of location with respect to disease etiology [14,15]. The concept of "activity spaces", the geographic areas within which people conduct their daily routines, offers a more complete way to assess the role of location on health. "Accessibility" is best understood in terms of activity space and multi-purpose trip making; not distance [16]. Destinations located within an individual's activity space are more likely to be patronized because they are more convenient and involve less travel [17]. The location of physicians relative to the location and size of activity spaces has been shown to influence health care utilization among the rural elderly [18]. However, activity spaces and access to transportation have been found to be more influential with respect to chronic care than acute care among the elderly [19,20]. In this study we use aggregate commuting patterns as a proxy for the activity spaces of rural residents.

Because both home and work locations are included in one's activity space, it is relevant to consider the role of commuting in health care seeking. However, the relationship between rural-urban commuting and health care seeking by rural residents has not been thoroughly investigated. Some authors have suggested that rural residents' trips to town for food and other services often serve as occasions for accessing health services [21] but trips to work are likely more important for two key reasons. First, long-time rural dwellers are increasingly working off-farm with regional urban places being the most frequent work destinations [22,23]. For this sub-population of rural people, accessing health care near their workplace may be particularly important because trips are usually scheduled at a convenient time unless the condition is perceived as life threatening [24]. Second, those who move from urban to rural places often retain many of their urban characteristics and so have strong social and economic ties to the urban environment [25].

A number of other general characteristics of people living in rural places affect health seeking. First, rural residents often postpone seeking care until it is economically or socially convenient [15]. Second, rural patients have a higher threshold for seeking care [14,15,26]. Rural parent's beliefs about pain management might be particularly relevant as mothers do not want to be perceived as weak or overanxious [27]. Third, rural residents may choose to bypass local providers for providers in urban settings. Surgical diagnoses, particularly, are associated with a greater chance of rural people bypassing local hospitals [28,29]. Ambulatory care admissions outside a patient's county of residence have been associated with younger age [30] and sociodemographic characteristics [31]. Finally, rural patients have more difficulty deciding what to do in the event of an emergency when they are unsure about the availability or accessibility of their primary care physician [32]. These health seeking behaviors may lead to delay in seeking care and suggest that rural populations with strong urban ties may be at higher risk of poor outcomes.

In this study, we test the hypothesis that children living in rural ZIP codes where the majority of people commute to an "urbanized area" (>50,000 people) have higher rates of perforated appendicitis than children living in rural ZIP codes where the majority of people commute to an "urbanized cluster" (10,000 – 49,999 people). Our rationale is that people in the former group would be more likely to bundle health care seeking with work trips – resulting in delay in receiving care and worse outcomes.

## Methods and Data

### Data

We used the Ohio Hospital Association (OHA) inpatient hospital claims extracts between January 2001 and December 2003 to perform a retrospective analysis of perforated appendicitis in children aged 2 to 20 years. The OHA receives data regarding all encounters relating to inpatient, outpatient, emergency department, and skilled nursing facility care at all hospitals in Ohio as well as hospitals bordering Ohio at which Ohioans receive care. The database for the period beginning January 1, 2001 and ending December 31, 2003 contained information about 8,274 pediatric appendectomies performed at 171 hospitals in Ohio. We included all patients with *International Classification of Diseases, 9*^*th *^*Revision Clinical Modification *procedure codes 470.1 (laparoscopic appendectomy) and 470.9 (other appendectomy) regardless of disease code. Perforations were identified as those patients with disease codes 540.0 (acute appendicitis with peritonitis) and 540.1 (appendiceal abscess). Patient age, sex, and ZIP code were available for each record (race was not available). Admission type, admission date, discharge date, discharge status, payer and total charges were also included in the database.

Of the 8,274 original cases, 188 were eliminated from the regression analysis leaving 8,086 observations. Of the 188 excluded cases, 120 were missing sex data, 49 had uncommon types of insurance (e.g. workman's compensation), and 19 had uncommon admission sources. Missing data eliminated all the cases at one hospital leaving 170 hospitals in the analysis.

We created a variety of variables categorizing places of residence and hospitals from the OHA data. We classified places of residence by rural-urban commuting area (RUCA) code using patient ZIP codes. The RUCA scheme classifies each ZIP code into one of thirty-three groups along the rural-urban continuum [6]. These codes are based on the population size of the ZIP code as well as the proportion of people who commute to a larger place for work. For example, RUCA code 10.1 defines an isolated rural ZIP code with no primary commuting flows over 5 percent to any Census Bureau defined Metropolitan Urbanized Area, Large Urban Place, or Small Urban Place *and *a secondary flow (30–50%) of people commuting to a Metropolitan Urbanized Area. We also created a binary rural variable which aggregates RUCA codes 10.1 and 10.4 into one category and 10.5 and 10.6 into a second category. Rural codes 10.1 and 10.4 identify ZIP codes where the majority of people commute from an isolated rural area to a metropolitan urbanized area. We refer to these ZIP codes as "Rural-UA". Rural codes 10.5 and 10.6 identify ZIP codes where the majority of people commuted from an isolated rural area to an urbanized cluster. We refer to these ZIP codes as "Rural-UC". Finally, ZIP codes of residence with RUCA codes designated as urban, large town, or small town were aggregated and referred to as "Non-Rural".

Hospital locations were classified as urban, large town, and small town based on the RUCA designation for the hospital ZIP code. We created this variable to capture potential differences in resource availability at hospitals in different locations. For example, differences in outcomes might arise from the lower availability of surgeons in small town settings. No hospitals were located in ZIP codes classified as isolated rural areas.

Seven hospitals were also classified as "children's" hospitals. We identified all freestanding, associate, and primary teaching children's hospitals in the database via the National Association of Children's Hospitals and Related Institutions (NACHRI) website [33]. These facilities have pediatric surgeons more likely to diagnose appendicitis quickly and correctly (particularly in the very young children) which could decrease the likelihood of perforation. Conversely, pediatric hospitals may be more likely to see complicated cases which could increase the likelihood of misdiagnosis and increase perforation rates. Also, parents might choose to bypass local care in favor of a children's hospital (located in a metropolitan area) if they perceive the quality of care to be higher.

Knowing the ZIP code of the patient and the ZIP code of the hospital permitted us to calculate approximate distances between patients' residence and hospitals. We used MapInfo 7.8 [34] to calculate the inter-ZIP code distances. We used distance (miles) between the center of the child's ZIP code and the center of the hospital ZIP code as an indicator of potential travel time to the hospital.

We controlled for comorbid conditions that might increase the severity of illness. In particular, we hypothesized that misdiagnosis or delay in diagnosing appendicitis would be more common patients with another infection or digestive disorder. A history of diarrhea is associated with increased risk of delay in diagnosis, especially in young children [35]. We created a binary variable coded "1" if any of the five ICD9CM diagnosis codes available in the OHA data included a code between 001 and 139.8 which includes all infectious and parasitic diseases. Similarly, we hypothesized that other intestinal/digestive problems or diseases would delay diagnosis of appendicitis. We created a binary variable coded "1" if any of the five diagnosis codes included a code between 520 and 537.9 or 550 and 579.9 (all digestive diseases excluding appendicitis). We also created binary variables for congenital disorders of the digestive system (codes 751 – 753.9) and pregnancy (630 – 669.9). Again we hypothesized that the presence of these conditions would delay diagnosis and increase the risk of perforation.

Finally, we obtained the USA 2000 Census data for Ohio at the ZIP Code Tabulation Area (ZCTA) level [36]. We used these data to calculate the appendectomy rate for all ZCTAs where the ZCTA population aged less than 21 years is the denominator and the number of appendectomies is the numerator. We also used the census data to calculate a deprivation index for each ZCTA. These data allowed us to model geographic differences in socioeconomic status by place of residence.

### Statistical Analysis

We used χ^2 ^analyses to test for differences in perforation rates across demographic, insurance, location, and comorbidity groups. We also used this statistic to test for differences in the rates of perforation at pediatric and non-pediatric hospitals. We used ANOVA to test for a difference in the mean distance traveled to hospital in Rural-UA commuting ZIPs versus Rural-UC commuting ZIPs.

We also performed a factor analysis (principal axis factoring with varimax rotation) of socioeconomic variables contained in Summary File 3 of the Census. Table 1 shows the census variables with significant (>0.40) factor loadings on the single factor extracted as well as the communalities and standardized coefficients. The SES factor extracted had an eigenvalue of 3.36 and a squared multiple correlation of 0.893. We derived factor scores for each ZCTA based on the standardized coefficients from this analysis.

**Table 1:** Factor analysis of socioeconomic status

Deprivation Index	Factor	Final	Standard
Variable	Loading	Communalities	Coefficients
Percent Single Parent Households	68	0.463	0.119
Percent no High School Education	72	0.516	0.170
Percent no vehicle available	75	0.562	0.180
Percent in Poverty	87	0.757	0.356
1 – percent employed (Percent Unemployed)	70	0.486	0.129
Median family income in 1999	-76	0.582	-0.203

We used a hierarchical, cross-classified, generalized linear model (HGLM) to calculate the multivariate odds of perforation [37]. We used this structure to control simultaneously for the clustering of cases within ZIP codes and OHA hospitals. Bivariate analyses were performed using SAS 9.1.3 [38]. The multivariate analysis was performed using HLM 6.06 [39].

This study received institutional review board (IRB) approval from the Nationwide Children's Hospital institutional review board.

## Results

### Bivariate analysis

Of the 8,086 children included in the analysis, the crude perforation rate was 25.5% (see Table 1). The χ^2 ^analyses revealed that perforation rates differed across age cohorts, sex, and insurance type. Children treated at children's hospitals were more likely to have perforated appendicitis than those treated at other hospital types (34.9% versus 22.4%, p < 0.001). Patients living in rural-UA ZIP codes were also more likely to have perforated appendicitis than those in rural-UC or non-rural ZIP codes (42.5% versus 27.1% and 25.4%, p = 0.042). There is a non-significant trend toward lower perforation rates in less urban locations with small town hospitals having the lowest perforation rates.

Comorbid conditions were also associated with higher rates of perforation. The perforation percentage among children with "other infections" (codes 001 – 139.8) was 66.6%. The percentage among children with "other digestive disease" (codes 520–537.9, 550–579.9) was 42.6%. In comparison, the overall rupture percentage was 25.4% (p < 0.001 in both cases). On the other hand, patients with congenital digestive diseases (codes 751 – 753.9) experienced perforation rates of 16.7% and pregnant females (codes 630 – 669.9) experienced perforation rates of only 6.9%.

**Figure 1:**
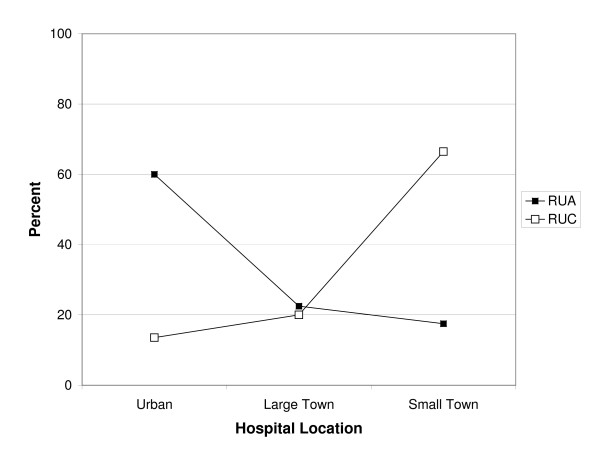
Hospital location choice by rural commuting category. (Black square) = Rural to urbanized area. (Open square) = Rural to urbanized cluster.

The hospital locations of patients living in rural-UA ZIPs compared to rural-UC ZIPs also differed. Patients living in rural-UA ZIPs were more likely to seek care in urban hospitals. In contrast, patients living in rural-UC ZIPs were more likely to seek care in small town hospitals (see Figure 1). The difference of proportions test is significant at p < 0.001. The average home-hospital distance traveled for Rural-UA patients was 25.5 miles and this compares to 9.2 miles and 9.1 miles for Rural-UC and non-rural children respectively (p < 0.001 for the ANOVA test in both cases). The 3 × 3 χ^2 ^test of dependence between place of residence (Non-rural, Rural-UA, Rural-UC) and hospital location (Urban, Large Town, Small Town) is associated with statistically significant differences in perforation rates (see Table 2).

**Table 2:** Patient Characteristics and Perforation Rates in OHA Hospitals

	Characteristic	n	Perforation %	sig.
Age & Sex	All	8086	25.5	
	Females 2–4	101	58.4	
	Females 5–9	550	30.5	
	Females 10–14	1095	26.8	
	Females 15–19	1548	14.7	
	Males 2–4	91	56.0	
	Males 5–9	839	34.8	
	Males 10–14	1813	28.5	
	Males 15–19	2049	22.3	p < 0.001

Insurance	Self	705	26.7	
	Medicaid	1460	27.7	
	Blue Cross Primary	840	26.7	
	HMO	1170	19.7	
	PPO	650	26.3	
	Medicaid HMO	223	34.5	
	Blue Cross HMO	101	16.8	
	Other	523	32.9	
	Private	2414	24.1	p < 0.001

Comorbidities	No other infection	7547	22.6	
	Other infection	539	66.6	p < 0.001
	No other digestive disease	7299	23.7	
	Other digestive disease	787	42.6	p < 0.001
	No digestive congenital anomaly	8026	25.6	
	Digestive congenital anomaly	60	16.7	p = 0.072
	Females not pregnant	3018	24.2	
	Females pregnant	276	6.9	p < 0.001

Patient & Hospital Combination	Non-rural ZIP to Urban Hospital	5986	26.0	
	Non-rural ZIP to Large Town Hospital	1483	24.3	
	Non-rural ZIP to Small Town Hospital	407	21.4	
	Rural-UA to Urban Hospital	24	45.8	
	Rural-UA to Large Town Hospital	9	44.4	
	Rural-UA to Small Town Hospital	7	28.6	
	Rural-UC to Urban Hospital	23	34.8	
	Rural-UC to Large Town Hospital	34	35.3	
	Rural-UC to Small Town Hospital	113	23.0	p = 0.050

	Children's Hospital	2052	34.9	
	Other hospital	6034	22.4	p < 0.001

### Multivariate Analysis

Table 3 shows the initial and final estimates of main effects. The null model (containing only intercepts for patient, residence ZIP category, and OHA Hospital) results show that both ZIP code of residence and OHA hospital are significantly associated with risk of perforation. Residence ZIP category accounts for approximately 3.0% of variation in perforation while OHA hospital accounts for approximately 16% of variation in perforation.

**Table 3:** Null and Full model main effects for patients, ZIP of residence and Hospital

Intercepts		Tau	% variance	P-value
Null model (intercepts only)				
Intercept – patients	theta0	0.964	0.812	0.000
Intercept – ZIP codes	b00	0.035	0.030	0.039
Intercept – hospitals	c00	0.188	0.159	0.000
Full Model (all level 1 & 2 covariates)				
Intercept – patients	theta0	0.973	0.867	0.000
Intercept – ZIP codes	b00	0.026	0.023	0.316
Intercept – hospitals	c00	0.124	0.110	0.000

Table 4 shows the results of the full multivariate analysis. Age and sex cohort effects are significant predictors of perforated appendicitis and follow the expected gradient. Controlling for age, sex, and comorbid conditions, insurance type is not significantly associated with perforation risk.

**Table 4:** Full Multivariate model

Covariates	Name	coefficient	Odds Ratio	95 lower	95 upper	P-value
**LEVEL ONE**						
Intercept	theta0	-2.043	0.130	0.096	0.174	0.000
Age & Sex						
Females 2 – 4	theta1	1.634	5.125	3.254	8.072	0.000
Females 5 – 9	theta2	0.639	1.896	1.475	2.436	0.000
Females 10 – 14	theta3	0.658	1.931	1.564	2.385	0.000
Females 15–20 (reference)			1			
Males 2–4	theta4	1.577	4.843	3.030	7.740	0.000
Males 5–9	theta5	0.919	2.508	2.013	3.125	0.000
Males 10–14	theta6	0.691	1.997	1.649	2.417	0.000
Males 15–20	theta7	0.532	1.703	1.413	2.053	0.000
Complications (binary)						
Other infection	theta8	1.889	6.611	5.392	8.106	0.000
Digestive Disorder	theta9	0.775	2.170	1.835	2.566	0.000
Pregnancy	theta10	-1.173	0.310	0.189	0.508	0.000
Congenital Digestive	theta11	-0.717	0.488	0.234	1.020	0.056
Insurance						
Medicaid insurance	theta12	-0.006	0.994	0.796	1.242	0.960
HMO insurance	theta13	-0.135	0.874	0.691	1.105	0.262
Other insurance	theta14	-0.023	0.977	0.740	1.290	0.870
Private insurance	theta15	-0.051	0.951	0.776	1.165	0.625
Self insured (reference)			1			
**LEVEL TWO**						
**ZIP code of residence**	b00					**0.316**
Deprivation index	G01	0.035	1.036	0.969	1.107	0.301
Appendectomy rate	G04	0.009	1.009	0.997	1.022	0.135
Rural – Urban Area	G02	0.737	2.090	1.038	4.209	0.039
Rural – Urban Cluster	G03	0.374	1.453	0.955	2.212	0.081
Other RUCA			1			
**OHA Hospital**	c00					**0.000**
Children's Hospital	B05	0.640	1.896	1.243	2.894	0.003
JCAHO accreditation	B01	0.152	1.164	0.942	1.438	0.158
Critical Access designation	B02	-0.009	0.991	0.708	1.388	0.959
Total number of appendectomies	B03	-0.001	0.999	0.998	1.001	0.347
Located in small town	B04	-0.150	0.861	0.607	1.22	0.400
Percent of app. with other infection	B06	0.000	1.000	0.989	1.012	0.985

Comorbid conditions are significant predictors of rupture. In particular, the odds of perforation are 6.61 (5.39, 8.12) for patients with other types of infections and 2.17 (1.84, 2.57) for patients with other digestive disorders. Congenital digestive anomalies and pregnancy are protective factors with odds ratios of 0.49 (0.234, 1.02) and 0.31 (0.189, 0.508) respectively.

Controlling for these comorbid conditions (and indirectly for differences in severity of illness), children who live in rural-UA ZIP codes have an adjusted odds of rupture of 2.09 (1.04, 4.21) compared to children living in non-rural ZIP codes. Though significant at only p = 0.08, children living in rural-UC ZIP codes have an adjusted odds of perforation of 1.45 (0.955, 2.21). Neither the appendectomy rate nor the deprivation index were significantly associated with perforation.

Among OHA hospitals, children whose surgery was performed at children's hospital had an adjusted odds of perforation of 1.90 (1.24, 2.89). The percentage of patients with other infections, JCAHO accreditation, critical access designation, location in a small town, and the number of appendectomies (volume) were not statistically significant. Smink et al. [9] also found no relationship between volume and rupture rates.

Table 3 shows the main effects of residence ZIP code category and hospital after including all covariates. Controlling for age, sex, and severity of illness, the fixed-effect coefficient for rural-UA remains significant. There is also a 5 percent reduction in the variance explained by OHA hospital after controlling for patient level covariates. Together, residential and OHA hospital effects are associated with 13% of the variation in perforation.

## Discussion

After controlling for age-sex cohort, comorbid conditions, and insurance type, we found that higher rates of perforation in Rural-UA areas remain statistically significant. This increased risk may be the result of bundling health care trips with regular work trips. Previous studies have found that delays as short as 12 hours and almost certainly after 48 hours are associated with increased incidence of perforation [40,41]. Parents may decide to take their child to a primary care provider, urgent care center or emergency room near their urban workplace if this is more convenient and/or feasible given work schedules. Similarly, parents might wait to seek care near their workplace if they judge the quality of care in urban hospitals to be higher. Familiarity with providers and hospitals near the workplace is another plausible explanation. Parents may wait until morning if they are simply unaware of facilities near their residence but know the location of services near their workplace.

While it is true that Rural-UA patients traveled 2.5 times farther than Rural-UC patients, the difference in distance is not likely to be an important component of the increased rupture rates. The mean distance traveled for Rural-UA patients was 25.5 miles and the driving time associated with this distance (approximately 30 minutes) is trivial in relation to the times considered significant in increasing risk of perforation (12–48 hours).

The results concerning comorbid conditions are not surprising because infections (e.g., *Escherichia coli*) may result in symptoms (e.g., diarrhea) that mask those of appendicitis; thereby delaying diagnosis of appendicitis. Further, these infections may be treated with antibiotics which would attenuate the symptoms of appendicitis. Similarly, the symptoms in patients having other digestive disorders may mask appendicitis and delay its diagnosis and treatment.

We originally hypothesized that congenital digestive anomalies and pregnancy would also delay diagnosis and treatment. It is possible that patients with congenital digestive anomalies are more aware of changes in the functioning of their digestive system and seek treatment sooner. Similarly, pregnant patients may seek treatment sooner when they are experiencing abdominal pain.

The results of this study have two important implications for service provision and clinician training aimed at reducing morbidity and costs associated with perforated appendicitis. Regarding services, DeLia [42] as well as Basu and Freidman [43] suggest that increasing primary care providers in underserved areas would be effective in reducing admissions for ambulatory care sensitive conditions (ACSC). Perforated appendicitis is an ACSC because timely care prevents perforation. However, the results of this study suggest that increasing resources near the *residences *of patients may not reduce ACSC admissions for perforated appendicitis if commuters bypass local care for providers that they are more familiar with, are located more conveniently, or perceive to be of higher quality. Primary care or other services should be located *conveniently *so as to reduce the propensity for delay in seeking care. Co-locating day surgery services near large rural retail stores might also be feasible (similar to dentists and optometrists). The next step for research in this area will involve detailed studies of activity spaces at the individual level to determine exactly where services should be delivered so as to be most attractive to the rural commuting population.

## Limitations

Our study has three primary limitations. First, we do not have clinical data or medical charts to include the actual or estimated time of delay in seeking care or delay in surgical consultation and diagnosis. Ideally, our model would include parents' estimated delay time, individual commuting habits, and place of residence in order to better link the three characteristics. Second, since the commuting data are only available at the ZIP code level, we are unable to connect individual parental activity spaces with individual differences in delay seeking care and the likelihood of perforation among children. Third, data regarding the race/ethnicity of patients was not available through the OHA database. Race is a known factor in the likelihood of perforated appendicitis [3,44], thus, some of our results may have been different had we been able to control for it.

## Conclusion

To our knowledge, this is the first study to differentiate rural dwellers with respect to outcomes associated with appendicitis as opposed to simply comparing "rural" to "urban". Risk of perforated appendicitis associated with commuting patterns is larger than that posed by several individual indicators including some age-sex cohort effects. After controlling for important individual level risk factors (age, sex, comorbid conditions), known contextual risks (neighborhood socioeconomic status), and hospital factors (volume, children's hospitals, accreditation) the statistical significance of commuting patterns persists. Further, the hospital locations where Rural-UA and Rural-UC patients sought care were consistent with the commuting patterns of the ZIP code in which they resided. Differences in rupture rates among rural children were not likely to be related to differences in distance traveled because the average distances traveled were short. Future studies linking the activity spaces of individuals to individual patterns of seeking care will further our understanding of perforated appendicitis and ambulatory care sensitive conditions in general. Activity spaces and multi-purpose trips that include health-related visits may be a promising avenue of future research into the health seeking behaviors of rural residents.

## Competing interests

The authors declare that they have no competing interests.

## Authors' contributions

RP had the original idea, conducted the data analysis and wrote the manuscript. DC assisted with data acquisition, planning the analysis and wrote sections of the discussion. BN assisted with planning the analysis and revising the manuscript. KK assisted with planning the analysis and revising the manuscript. RP is the guarantor.
